# Exercise intolerance with ageing: Major role for vascular dysfunction?

**DOI:** 10.1113/EP091606

**Published:** 2023-12-06

**Authors:** David C. Poole, Glenn A. Gaesser

**Affiliations:** ^1^ Department of Kinesiology Kansas State University Manhattan Kansas USA; ^2^ College of Health Solutions Arizona State University Phoenix Arizona USA

**Keywords:** aging, critical power, exhaustion, healthspan, muscle function, vascular control

1

Our health, happiness and, to a certain extent, our lifespan link inextricably – but not inexplicably – to our capacity for exercise and physical activity. Hippocrates opined ‘If we could give every individual the right amount of nourishment and exercise … we would have found the safest way to health’, and today cardiorespiratory fitness, defined as maximal oxygen uptake (${\dot{V}}_{O_{2}\max}$), is recognized as the best predictor of all‐cause mortality. Notwithstanding this, critical power (CP, or its running equivalent, critical speed, CS), which defines the boundary, and metabolic rate, between heavy (submaximal ${\dot{V}}_{O_{2}}$) and severe (yielding ${\dot{V}}_{O_{2}\max}$ at exhaustion) intensity exercise often has a stronger relationship with athletic performance and setting optimum training loads than ${\dot{V}}_{O_{2}\max}$ (Poole et al., 2021). Put another way CP (or CS) defines the boundaries of human performance for high intensity exercise and, as such, may be a superior arbiter of functionality and healthspan.

As we age, exercise capacity, defined as CP/CS, decreases progressively attended and likely caused by some combination of reduced cardiovascular and muscle function. In an article in this issue of *Experimental Physiology*, Dorff et al. (2023), in modest cohorts of active (∼13,000 steps per day, ∼2.5 times US average) young (mid‐20s) and older (60s) men and women, tested the hypothesis that, when accounting for any differences in muscle mass, the age‐related decline in CP would be related to impaired vascular function and reduced muscle blood flow. To focus more directly on skeletal muscle rather than cardiac limitations per se, Dorff et al. (2023) utilized knee‐extension exercise and assessed vascular function using their single passive leg movement technique that evokes a heavily nitric oxide‐dependent hyperaemia.

It may rightly be argued that when a process such as ageing degrades an entire system and reduces the criterion work rate, the expectation is that decreases in CP and blood flow would be correlated, and this is what Dorff et al. (2023) did indeed demonstrate. What adds to the importance of this observation is that, even at the same sub‐CP work rate of 20 W, the leg extensor blood flow in the older, compared with the younger, subjects was decreased by ∼30% and this was correlated significantly with the passive leg movement assessment of endothelial function, identifying a vascular target for future studies aimed at developing effective countermeasures to age‐related declines in CP. Specifically, unless the metabolic cost of the exercise was 30% lower in the older subjects – and such is highly doubtful (see Gaesser et al. (2018) for no difference for metabolic cost (${\dot{V}}_{O_{2}}$) of cycling or walking in older versus younger adults) – a decreased O_2_ delivery‐to‐${\dot{V}}_{O_{2}}$ ratio will reduce microvascular, interstitial and probably intramyocyte O_2_ partial pressures, compromising O_2_ diffusion and mitochondrial energetics regulation (Poole et al., 2021).

Dorff et al. (2023) rightly caution against direct application of leg‐extension to whole body exercise such as walking, running or cycling, for example. But it is notable that leg‐extension based exercise training programmes in older heart failure patients have translated to improvements in conventional whole‐body cycling performance (Esposito et al., 2011). Accordingly, what if age‐related decrements in vascular function reduce CS such that this limits the speed at which the elderly walk? Could this help explain, and indeed be mechanistically responsible for, the association between self‐reported walking pace and 10‐year cause‐specific mortality (fast versus slow walkers, significantly reduced hazard ratios for CVD, cancer and other causes) extracted from 391,652 UK Biobank participants by Goldney et al. (2023)?

Dorff et al.’s findings thus set the imperative for mechanistic studies seeking to test whether experimental treatments, targeting vascular control specifically (i.e., a target upstream from the myocytes), can help reverse the age‐related decline in CP/CS. The capability to increase walking speed, especially in the elderly, would likely pay dividends in elevating quality of life and extending the health‐span and is eminently deserving of attention.

## AUTHOR CONTRIBUTIONS

Both authors have read and approved the final version of this manuscript and agree to be accountable for all aspects of the work in ensuring that questions related to the accuracy or integrity of any part of the work are appropriately investigated and resolved. All persons designated as authors qualify for authorship, and all those who qualify for authorship are listed.

## CONFLICT OF INTEREST

The authors declare no conflicts of interest.

## FUNDING INFORMATION

No funding was received for this work.
